# Synthetic soils for ecological and synthetic biology applications

**DOI:** 10.1093/femsre/fuag012

**Published:** 2026-03-20

**Authors:** Jack Orebaugh, Alyssa A Carrell, Larry M York, Melissa A Cregger, Ilenne Del Valle Kessra

**Affiliations:** Biosciences Division, Oak Ridge National Laboratory, 1 Bethel Valley Rd, Oak Ridge, TN 37831-6038, United States; Biosciences Division, Oak Ridge National Laboratory, 1 Bethel Valley Rd, Oak Ridge, TN 37831-6038, United States; Biosciences Division, Oak Ridge National Laboratory, 1 Bethel Valley Rd, Oak Ridge, TN 37831-6038, United States; Biosciences Division, Oak Ridge National Laboratory, 1 Bethel Valley Rd, Oak Ridge, TN 37831-6038, United States; Biosciences Division, Oak Ridge National Laboratory, 1 Bethel Valley Rd, Oak Ridge, TN 37831-6038, United States

**Keywords:** soils, standardized habitats, synthetic biology, ecology, artificial soils

## Abstract

Soils are heterogeneous and dynamic systems characterized by complex physical, chemical, and biological interactions. Understanding these interactions is critical, as they influence plant productivity, global biogeochemical cycles, and ecosystem resilience. While ecologists have long studied soils in field, greenhouse, and laboratory settings, their complexity and heterogeneity make it challenging to pinpoint key properties driving biological processes and derive mechanistic insights. Advancements in synthetic biology, which seeks to engineer and control biological processes in soils, have increased the demand for standardized and controllable experimental platforms. These platforms, referred to here as ‘synthetic soils’, are systems designed to reproduce selected physicochemical characteristics of natural soils in a simplified and defined format, allowing scientists to systematically change soil physicochemical properties (i.e. texture, mineralogy, pH) to study how biological components (i.e. microbes, plants, soil fauna, etc.) respond to, modify, or interact within these controlled environments. This review explores existing synthetic soils, their advantages, limitations, and applications in ecology and synthetic biology, and discusses potential directions for their future development.

## Teaser

This review summarizes recent advances in the development of standardized synthetic soils to study how soil physicochemical properties shape plant-microbe interactions for applications in ecological research and synthetic biology.

## Introduction

Soils are dynamic and heterogenous systems composed of minerals, organic matter (OM), water, and air. Their physical structure and chemical composition create habitats for diverse soil microbes, flora, and fauna and contribute to the incredible diversity that can be found across the globe (Vos et al. 2013, Fierer 2017, Zengler et al. 2019, Smercina et al. 2021). Soils mediate biogeochemical cycling and are fundamental to ecosystem productivity, climate regulation, and agricultural sustainability (Vos et al. 2013, Fierer 2017, Zengler et al. 2019, Smercina et al. 2021). Understanding how soils function is essential for improving land management and agricultural practices, and predicting ecosystem responses to global change (Trivedi et al. 2020, Kleber et al. 2021, Jansson et al. 2023).

For decades, soils have been studied to characterize their physical, chemical, and biological composition (Trivedi et al. 2020, Kleber et al. 2021, Jansson et al. 2023), using a range of experimental approaches, each suited to addressing different types of questions. Field-based studies and experiments are essential for capturing natural heterogeneity, spatial structure, and emergent ecological dynamics in soils. Laboratory and greenhouse experiments using field-collected soils provide greater experimental control while retaining much of the native biological and physicochemical complexity. However, both approaches remain constrained by the intrinsic spatial heterogeneity and limited tunability of natural soils. Because mineral composition, organic matter chemistry, pH, and soil water retention co-vary, manipulating one property necessarily alters others, restricting the ability to isolate mechanisms or impose defined boundary conditions in the experiments.

Moreover, many plant-microbe-soil interactions take place at spatial and temporal scales that are poorly resolved using field soils. Microbial activity responds to short-lived inputs such as root exudates or rainfall, creating transient ‘hot-moments’ of gene expression that impact growth and biogeochemical transformations within minutes to hours and are often missed by destructive or low-frequency sampling (Kuzyakov and Blagodatskaya 2015, Del Valle et al. 2021). At the same time, soils have micron scale heterogeneity in pore connectivity, hydration, and diffusion that creates microhabitats where cells are separated into localized neighborhoods with sharp chemical and oxygen gradients (Smercina et al. 2021). Within these microhabitats, enzyme activities are impacted by the bioavailability of substrate and co-factors, while biofilm formation and physical confinement affects cell-to-cell contact and processes such as horizontal gene transfer (HGT) (Gillett et al. 2025). Yet major unknowns remain regarding how soils regulate enzyme activity, as well as how genes are transferred within soil microbiomes and how soils control this exchange.

**Figure 1:**
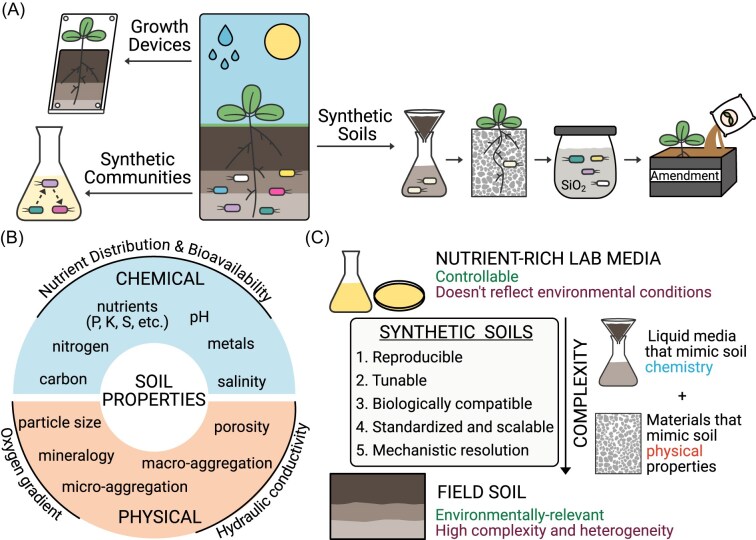
Experimental frameworks and design principles for synthetic soil systems. A) Experimental platforms used to simplify complex soil environments. Controlled growth devices (e.g. EcoFABs, Ecotrons, etc.) regulate environmental parameters such as hydration, temperature, and light. Standardized synthetic microbial communities, composed of two or more defined, culturable species that stably persist in well-defined media, reduce biotic complexity. Synthetic soils, consisting of simplified materials that mimic key soil properties, can be combined with growth devices and synthetic communities to independently manipulate abiotic soil factors. B) Soil properties that influence biological processes and should be recapitulated in synthetic soils in a controllable manner. Chemical properties include macro- and micronutrients, pH, metal availability, and salinity. Physical properties include particle size distribution, mineralogy, porosity, and aggregation. C) Conceptual positioning of synthetic soils along a continuum between homogeneous, nutrient-rich laboratory media and complex field soils. Ideal synthetic soils are reproducible, biologically compatible, scalable for larger-format experiments (e.g. supporting larger plants) as well as for high-throughput applications requiring large sample numbers, and tunable to enable mechanistic resolution of biological processes. No single system achieves all these characteristics; rather, existing approaches span a spectrum from liquid media designed to approximate soil chemistry to structured materials and devices that replicate key physical properties of soil.

To address these limitations and improve experimental control and reproducibility, researchers have developed a range of simplified experimental systems that reduce biological and environmental complexity. These include assemblies of isolated bacteria and fungi with reduced complexity, also known as synthetic microbial communities (SynComs) (Northen et al. 2024), as well as standardized plant and microbe growth chambers, such as ecotrons, rhizoboxes, and fabricated ecosystems (EcoFABs), which enable control over environmental parameters (e.g. light, water, temperature, humidity) and compatibility with sensors (e.g. O₂, CO₂) (Fig. 1A) (Sasse et al. 2019, Roy et al. 2021, Kelliher et al. 2024, Northen et al. 2024, Novak et al. 2024a, 2024b).

Another approach to simplification focuses on the soil matrix itself. Synthetic soils are simplified and controllable experimental platforms designed to systematically replicate specific soil properties (e.g. pH, texture, porosity) and are positioned along a continuum of experimental systems, more environmentally representative than homogeneous rich media, yet more experimentally tractable and less complex than field soils (Fig. 1C). By independently manipulating these properties, researchers can test mechanistic hypotheses and establish causal relationships between defined soil parameters and biological responses, disentangling variables that are inherently coupled in natural soils. These systems are useful for studying biological processes across various spatial scales (µm to cm) and temporal dynamics. They can be designed to be compatible with analytical and imaging tools that are difficult to apply in opaque, heterogeneous natural soils. They can also be integrated with defined microbial communities (e.g. SynComs) and standardized growth platforms to further enhance reproducibility and cross-study comparability (Mair et al. 2025). The purpose of synthetic soils is not to replace field sampling or experiments using field soils, but rather to complement these approaches by enabling controlled experimentation for questions that require reduced environmental variability and precise manipulation of soil parameters that are otherwise inseparable in natural systems.

The characteristics of an “ideal” synthetic soil depend on the specific application and research question, and no single synthetic soil is expected to capture the full complexity of natural soils. In general, these systems should reproduce the soil physical and chemical properties of interest within defined and ecologically relevant ranges (for example, soil water retention values observed in natural soils). Soil properties that influence biological function and are of interest to mimic in synthetic soils include physical properties such as soil structure (macro- and microaggregation), texture, porosity, and mineralogy (Vos et al. 2013). Relevant chemical properties include primary macronutrients (carbon, nitrogen, phosphorus, potassium); secondary macronutrients (calcium, magnesium, sulfur); micronutrients; metals; salts; and pH (Fig. 1B) (Philippot et al. 2024). Importantly, carbon and nitrogen should not be considered solely in terms of total concentrations, but also in terms of the chemical complexity, molecular composition, and bioavailability of soil organic matter (SOM), including factors such as functional group chemistry, degree of polymerization, and mineral association, all of which strongly regulate microbial activity and nutrient cycling (Lehmann and Kleber 2015a, Schmidt et al. 2011). Although these soil properties are known to shape plant performance and microbiome composition (reviewed in Bennett and Klironomos 2019, Philippot et al. 2024), the mechanistic bases underlying these effects remain poorly understood. In particular, we lack predictive frameworks that link individual and co-varying soil properties to signaling dynamics, nutrient exchange, and microbial spatial organization; that quantify how microbially driven modifications of soil structure and chemistry feedback on community interactions; and that explain how plant-soil feedback shift across physicochemical contexts through changes in nutrient bioavailability, hydraulic connectivity, and gas diffusion.

Synthetic soils should be designed such that the property of interest can be varied independently of other properties, allowing the effects of specific variables to be isolated from confounding factors; for example, texture can be varied while maintaining constant pH or mineralogy. Additionally, the production of synthetic soils should be robust and reproducible to ensure consistency across experiments and laboratories. When feasible, scalability and compatibility with high-throughput experimentation are important for translating insights from laboratory studies to mesocosm or greenhouse scales and for generating datasets suitable for data-driven and machine-learning approaches. If used for plant studies, synthetic soils must also be sufficiently stable to support plant growth over relevant experimental time scales. Finally, compatibility with analytical tools is an important design consideration; matrix composition can influence both imaging and molecular analyses, as opaque or light-scattering components may limit imaging and certain minerals, such as clays, can impede genetic material extraction and downstream omics analyses (Nunan et al. 2020, Zhang et al. 2025).

A well-designed synthetic soil can be used to study ecological questions aimed at understanding natural ecosystem processes supported by soils, as they can be compatible with measurements beyond traditional soil microbiome metrics, such as bacterial-to-fungal ratios or phylum-level abundances (Ossowicki et al. 2021, Mishra et al. 2023). By systematically varying specific soil parameters, these systems provide mechanistic information on how individual soil properties influence the soil microbiome, including environmental filtering, niche partitioning, coexistence, community assembly rules, and diversity-stability relationships, that affect microbial abundance, structure, and interactions (Baran et al. 2015, Laland et al. 2016). Synthetic soils can also be used to determine how soil characteristics influence plant performance, including growth, nutrient acquisition, and stress resilience, as well as how microbes and plants actively modify soils through processes such as nutrient cycling, mineral weathering, microbially induced carbonate precipitation, extracellular polymeric substance production, metal chelation, and changes in root structure.

In addition to ecology research, synthetic soils can support synthetic biology applications, a multidisciplinary field that applied engineering principles to redesign or introduce new functions into living systems (Clauer et al. 2026). These systems can be used for prototyping engineered organisms or communities designed to improve growth and stress tolerance of plant and microbes for bioenergy and agricultural applications, enable bioremediation, biosensing of chemicals and contaminants, biomining, and promote ecosystem services, such as carbon sequestration (Fierer 2017, Zengler et al. 2019, Zhalnina et al. 2021, Fierer and Walsh 2023, Jansson et al. 2023, Jones et al. 2024), and evaluating their fitness, adaptation, and gene circuit robustness of the engineered systems prior to trials in natural soils. Furthermore, these systems can be used to study plant-microbe interactions, including microbial communication, root exudation patterns, rhizosphere modification, and other interactions involving engineered plants and microbes.

Although the use of synthetic soils is gaining traction, most reviews focus on a single type of system (e.g. Aleklett et al. 2018) rather than synthesizing the range of synthetic soils, their applications, and their relevance to ecological and synthetic biology research (Mair et al. 2025). Synthetic soil systems span a gradient of structural, chemical, and biological complexity, ranging from chemically defined liquid extracts lacking physical structure, to structured transparent and microfluidic systems enabling microscale visualization, to aggregated artificial soils and amendment-based substrates compatible with greenhouse-scale experiments. Here, we summarize the current state of synthetic soils, highlighting their advantages, disadvantages, and prior applications, many of which remain at the proof-of-concept stage, and discuss potential ecological and synthetic biology questions they can address (Table 1). We classify these systems into three categories: chemical property replication, physical property replication, and synthetic soils that are, or could be, scaled to greenhouses.

## Synthetic soils for studying chemical properties

Soils consist of both inorganic and organic components. Understanding soil’s chemical composition, properties, and the potential for chemical reactions at various spatiotemporal scales is key to assessing their impact on plants and microbes (Sparks 2019). Traditionally, studies aiming to isolate the impact of chemistry on plant and microbial interactions have used standardized media such as minimal media like M9 and R2A for microbes, or Hoagland’s and Murashige and Skoog media for plants (Schierstaedt et al. 2020, Shrestha et al. 2021). As technology continues to improve and methods continue to be developed for capturing higher-resolution chemical data, it is crucial to study the chemical landscape of soils. Two promising additions to chemical studies are soil-extracted solubilized organic and inorganic matter (SESOM) and metabolomics-informed media (MIM). Although these systems are not soils in a structural sense, as they lack aggregation and physical complexity, they are derived from soil parameters and can reproduce environmentally relevant chemical conditions. As such, they provide greater ecological relevance for experimentation compared to traditionally used rich media in the laboratory.

### Soil-extracted solubilized organic and inorganic matter

Soil-extracted solubilized organic and inorganic matter (SESOM) is a type of liquid extract from field soils that preserves the chemical environment of an environmental sample (Liebeke et al. 2009). Natural soils are passed through a series of washing and filtering steps to collect the soluble fraction of metabolites without noise from other environmental factors, such as particulate organic carbon or plant residues, that could influence experiments (Liebeke et al. 2009). SESOMs require commercially available or field soils for chemical extraction, and they can be an ideal base system for isolating novel environmental microbes and testing hypotheses about the chemical effects on the growth and behavior of different organisms (NandaKafle et al. 2018). SESOMs contain both low and high molecular-weight organics, inorganic compounds, carbohydrates, and if desired, they can be adapted to include the natural soil microbiome by increasing filter size (Lehmann and Kleber 2015b, Liebeke et al. 2009). SESOMs offer researchers a cost-effective method for producing chemical-based liquid media using natural soils. However, since SESOMs are sourced from these natural soils, variations can occur between each extracted sample, and analyzing these chemical profiles still requires specialized analytical equipment (Manktelow et al. 2021, Yaacob et al. 2021).

**Figure 2:**
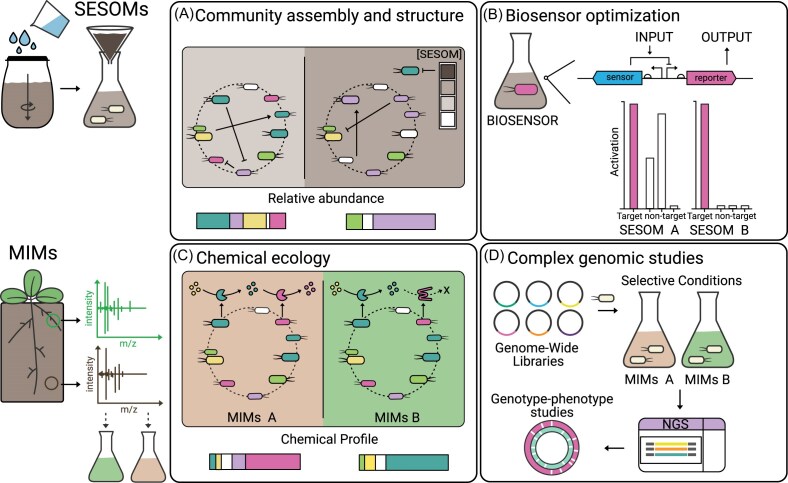
Liquid media formulations designed to mimic soil chemical properties. Soil-extracted solubilized organic and inorganic matter (SESOMs) (A, B) and metabolomics-informed media (MIMs) (C, D) represent chemically defined systems that approximate aspects of soil chemistry while maintaining experimental control. A) SESOMs can be used to investigate microbial community assembly and structural dynamics under soil-relevant chemical conditions. B) SESOMs provide a platform to prototype and evaluate biosensors, including assessing signal crosstalk and specificity to target inputs. C) MIMs enable studies of chemical ecology, such as shifts in metabolite profiles resulting from differences in enzymatic activity or the addition of specific bacterial species. D) MIMs can be used to benchmark complex datasets (e.g. genome-wide libraries) by precisely controlling the identity and concentration of metabolites added to the medium.

SESOMs have been used to characterize microbial physiology and behavior, including growth rates and metabolite production, and to study plant-microbe interactions, such as how root exudates influence microbial communities (NandaKafle et al. 2018, Manktelow et al. 2021, Sandhu et al. 2021). Looking forward, SESOMs are excellent platform to monitor the survival of a single microbe over time, the stability of microbial communities, positive and negative interactions following the introduction of a helper strain, and the impact of abiotic perturbations (e.g. changes in temperature) (Fig. 2A). They could also be used to screen for novel biosynthetic pathways activated by soil-derived metabolites.

SESOMS can also be useful in synthetic biology applications, including characterizing the growth of engineered strains and evaluating crosstalk and the specificity of sensor systems (e.g. transcriptional regulators) caused by the presence of metabolites in the soil (Fig. 2B). Furthermore, SESOMS provide a platform for assessing the availability and accessibility of substrates necessary for producing metabolites of interest that have been engineered into a host organism. They can help explore how chemical feedback mechanisms influence gene expression and the performance of engineered traits, as well as the tolerance of these traits to fluctuations under various nutrient levels (Hansen et al. 2025;). Finally, SESOMs could be used to assess interspecies interactions within synthetic communities when exposed to complex nutrient sources.

While SESOMs help ascertain chemical profiles from natural soils, they can be difficult to replicate due to the inherent variability in soil sample composition. Differences in extraction efficiency and the preference for metabolites in the aqueous phase can also introduce biases. These challenges make it difficult to achieve consistent results, as no two extractions from natural soil samples will yield the same outcome. This variability can hinder data normalization and analysis of treatment groups, as the basal media may not be as homogeneous within treatment groups compared to other methods. Furthermore, the high variability in the chemical composition of SESOMs makes it difficult to modify the recipe, such as removing a specific metabolite to assess its impact on microbial processes.

Modifying SESOMs by tailoring extraction methods (e.g. aqueous vs. other solvents) can enrich specific compound classes without sacrificing chemical diversity. Standardizing SESOM extraction methods could support broader adoption in the scientific community. Furthermore, as high-resolution analytical techniques advance, they will provide greater insight into the composition of these complex metabolite mixtures. For example, utilizing advanced chemical analyzers, such as a Fourier Transform Infrared Mass Spectrometer (FTIR-MS), can enhance the resolution and identification of SESOM composition (Mehmood et al. 2019).

### Metabolomics informed media

Metabolomics-informed media (MIM) are complex microbial growth media designed to replicate the complex chemical environment found in soils by leveraging cutting-edge exo-metabolomics. They represent a promising step toward recreating soil chemical conditions in a reproducible manner that is compatible with other routine laboratory analyses. (Jenkins et al. 2017). Technologies like Liquid Chromatography Tandem Mass Spectrometry (LC-MS/MS), Nuclear Magnetic Resonance (NMR), and other analytical chemistry techniques can provide high-resolution chemical data from diverse soil samples (Patyra et al. 2022). This chemical data can be used to identify key metabolites and their concentrations to create a medium recipe containing major compound classes such as sugars, organic acids, and amino acids. This recipe can be prepared using commercially available standards, ensuring consistent and reproducible results. MIM can be used as liquid or solid media for growth. Its easy formulation allows for the addition or removal of individual metabolites of interest to test their effects on plant or microbial growth. The media usually contains a mix of essential cofactors, such as vitamins and minerals, and metabolites derived from the metabolomics studies with variable C:N ratios consistent with soil biomass (Jenkins et al. 2017). MIMs provide an opportunity to study chemical interactions without the noise of other confounding factors, such as large recalcitrant organic compounds.

Numerous studies have shown that soil exo-metabolites strongly affect overall microbial abundance and community structure and functioning (Baran et al. 2015, Swenson et al. 2015). By exerting selective pressure on microbial communities, soil exo-metabolites also alter microbial metabolite production over time (Swenson et al. 2018). This cyclical property of exo-metabolites makes them increasingly important for understanding soil environmental processes over time (Swenson et al. 2018). For this reason, MIMs have been frequently used to study different physiological responses of organisms to various soil and/or plant metabolites, generally coupled with pre- and post-growth mass spectrometry analysis to evaluate changes in metabolite profile composition (Swenson et al. 2015, 2018). MIMs have also been specifically designed to promote the growth of targeted microbial strains or to facilitate the isolation of previously unculturable microbes (Jenkins et al. 2017).

MIMs can be a useful model system to answer questions in chemical ecology, such as the effects of adding various chemicals, including contaminants, antibiotics, or signaling molecules (Fig. 2C). They can also facilitate the formulation of new simplified bacterial or fungal communities to study coexistence, competition, and synergistic interactions (Chen et al. 2024). By adding complex carbon sources (e.g. cellulose, lignin), researchers can identify microbial degradation patterns and key OM decomposers. Introducing limited phosphorus or nitrogen to the MIM can simulate nutrient-limitation stress and provide insights into microbial strategies for nutrient acquisition (Wang et al. 2022). Finally, by adjusting the MIM to reflect root exudates from specific plant species, researchers can explore how plant genotypes shape their associated microbiomes.

MIMs can also be used to characterize plant and microbial systems engineered through synthetic biology. For engineered plants, MIMs can serve as a growth medium to analyze the production and exudation of engineered root metabolites under varying substrate availability (Griffin et al. 2024). For engineered microbes and microbial communities, MIMs can help assess survival and stability in the presence of different nutrient sources (Jenkins et al. 2017). Additionally, they can be employed for more complex studies, such as microbial genome-wide libraries (e.g. random barcode transposon-site sequencing (RB-TnSeq) or CRISPR interference (CRISPRi) libraries) to facilitate genotype-to-phenotype discoveries (Fenster and Eckert 2021) (Fig. 2D). Their defined environment simplifies downstream analysis by enabling clearer correlations between factors and library performance.

While MIMs can be suitable for specific applications, it is important to understand their limitations. MIMs provide a narrow experimental design window, as the media does not consider any physical characteristics of soils. The physical structure of soils modulates the accessibility of water and metabolites to plant roots and microbes by altering the physical distribution of the resources or through processes like sorption to the mineral phase, which changes bioavailability (Kleber et al. 2021). These dynamics result in specialized niches and hotspots, contributing to the complexity and variability of soil processes. In addition, MIMs provide a simplified representation of a natural soil chemical environment, as metabolomic analyses may miss low-abundance or transient metabolites that are essential for microbial growth, and some metabolites may be expensive or difficult to obtain. Furthermore, adding chelators or stabilizers to the media can alter the media’s chemical composition compared with a natural sample (Jenkins et al. 2017). MIMs also require careful and high-resolution metabolomic analysis for their creation (Swenson et al. 2015, Nemadodzi et al. 2020). This can impose significant research costs, as high-resolution mass spectrometry is expensive and requires careful sample preparation to reduce contamination. In addition, the limited generalizability of a specific MIM recipe further limits its accessibility and broader adoption for ecological and synthetic biology applications. Developing an online database of exo-metabolomic data derived from diverse bulk soils and plant rhizospheres could support MIM-based experiments. This resource would encourage the adoption of MIMs over traditional nutrient-rich media or alternatives like R2A, facilitating iterative testing and collaboration within the scientific community. As -Omics capabilities continue to improve and the need for chemically informed substrates continues to be relevant, MIMs will be an invaluable tool for understanding and harnessing the chemical environment within soils.

## Synthetic soils for studying physical properties

Soil physical characteristics, from micron-scale pores to macro-scale aggregates, create distinct micro- and macro-environments that provide niches for organisms and the structural framework for biogeochemical interactions within soils. (Smercina et al. 2021). Due to the importance of these physical environments, researchers across disciplines have devised various standardized soil systems to recreate specific physical properties for studying plant-soil-microbial interactions (Yee et al. 2021). One of the most attractive properties designed into these synthetic soil systems is their compatibility with optical measurements, allowing visualization of micro-scale biological processes with high resolution. The most prominent approaches are transparent soils and microfluidics that mimic soils properties, notably Rhizosphere-on-a-chip devices (Downie et al. 2012, Zhu et al. 2022).

### Transparent soils

Transparent soils (TS) consist of solid or semi-solid particles made from materials with a low refractive index (RI) or spherified gelling agents. They support unrestricted root growth in three-dimensional (3D) space and provide porosity for studying fluid flow and granular material behavior, while enabling optical imaging of root and microbial dynamics (Downie et al. 2012, 2014, Kuijken et al. 2015, Yuan et al. 2019, Liu et al. 2020, Ma et al. 2023, Sotta et al. 2025). Among the various TS developed, the two most commonly used are Nafion and hydrogels (Kuijken et al. 2015, Ma et al. 2019, Sharma et al. 2020, Li et al. 2022).

One of the earliest TS systems was developed using Nafion, a sulfonated tetrafluoroethylene-based fluoropolymer-copolymer. This material has a refractive index (RI) similar to that of water and becomes transparent when fully hydrated (Downie et al. 2014). Nafion can be adapted to have different properties, such as porosity and CEC. It is autoclavable, chemically stable, and compatible with fluorescence microscopy, tomography, and other optical methods (Downie et al. 2012, Rooney et al. 2024). Later, spherified hydrogel beads were developed as an alternative TS material. These beads can be formed by dropping a solution of gellan gum and alginate into a stirred MgCl₂ solution, or by crosslinking low-acyl gellan gum and hydroxyethyl cellulose with magnesium ions (Ma et al. 2019, Xie et al. 2024). They are inexpensive to produce, and their size and hardness can be tuned to mimic specific aspects of soil aggregation. Hydrogel spherification has enabled two key applications: (1) encapsulating beneficial microbes for delivery into soils, and (2) mimicking soil aggregate particles to support plant and microbes in a transparent, three-dimensional structured soil-like environment.

**Figure 3:**
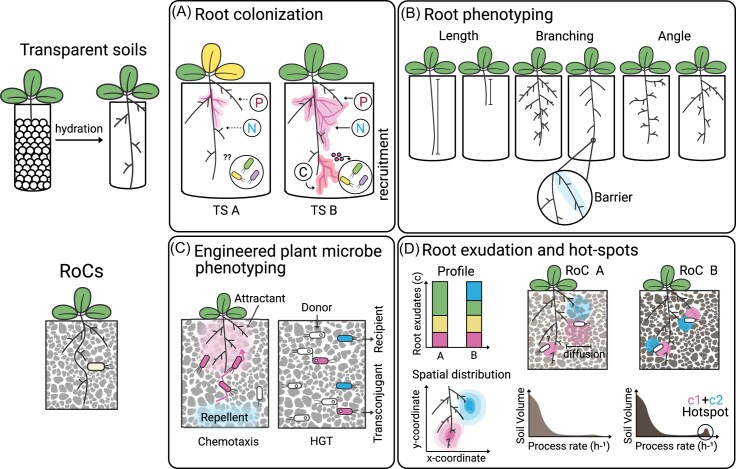
Transparent platforms for visualizing soil and rhizosphere processes. Transparent soils (TS) (A, B) and Rhizosphere-on-a-Chip systems (RoCs) (C, D) enable direct imaging and spatially resolved analyses of plant–microbe interactions. A) TS allow visualization of microbial colonization dynamics and bacterial recruitment along roots under controlled conditions. B) Transparent soils provide a platform to prototype engineered plants with altered root traits, including root length, branching patterns, growth angle, and the formation of root barriers (e.g. suberin and lignin deposition). C) RoCs are compatible with fluorescence imaging and can be used to track engineered microbes with defined functions, such as engineered chemotaxis or horizontal gene transfer. D) RoCs are also compatible with analytical techniques (e.g. MALDI imaging), enabling spatial mapping of root exudation patterns and identification of chemical hotspots where process rates are elevated.

TS are compatible with a variety of optical technologies, including tomography, optical cameras, microscopy, and fluorescence. They have been primarily used to study plant root growth and architecture, spatial imaging of root exudates, and the colonization and distribution of fluorescently tagged bacteria along roots (Downie et al. 2012, Yuan et al. 2019, Mathes et al. 2020, Jones et al. 2021, Jiang et al. 2022, Lozano-Andrade et al. 2023, Ma et al. 2023). Imaging through the matrix does not require sample disruption, making it suitable for plant-microbe time-series experiments, including studies of nutrient and carbon exchange, plant microbiome assembly, root colonization, and functional responses to stimuli (Fig. 3A) (Downie et al. 2014, van Veelen et al. 2018, Liu et al. 2020). TS are also valuable for computer vision applications, helping to train algorithms for image segmentation and classification (Massalha et al. 2017, Bai et al. 2023, Diwan et al. 2023, Liu et al. 2024).

TS are also effective for prototyping engineered plant-microbe interactions. For example, they can be used to phenotype engineered plant root structures (Fig. 3B), to study colonization by strains modified with plant growth-promoting traits, and to facilitate interkingdom synthetic communication (van Veelen et al. 2018, Essington et al. 2024). Additionally, hydrogel beads can be adapted for controlled nutrient release, which is valuable for studying ecological hot spots or for implementing engineered systems with logic-gate functions that require multiple inputs to trigger a response. They can also support memory encoding via recombinases or CRISPR systems that report on transient environmental events (Essington et al. 2024).

Despite their advantages, TS have limitations. Nafion is expensive, stains easily, and can induce osmotic stress due to the high osmolyte concentrations needed for RI matching (Rooney et al. 2024). It also fails to replicate larger soil aggregates, as increasing particle size reduces optical clarity (van Veelen et al. 2018, Essington et al. 2024). Hydrogels, though cost-effective, suffer from lower mechanical strength and are prone to dehydration, leading to shrinkage and compaction that compromise long-term stability (Kandemir et al. 2018).

To address these limitations, researchers are developing new hydrogel materials with enhanced crosslinking to improve structural integrity and reduce dehydration (Zhang and Khademhosseini 2017). Advances in semi-automated preparation systems are also improving manufacturing consistency of TS (Sotta et al. 2025). A combination of materials with different stiffnesses and sizes, or the incorporation of transparent additives such as glass beads or aerogels, could help simulate natural particle distribution and porosity to better mimic natural soil (Zhang and Khademhosseini 2017, Kandemir et al. 2018).

Recent advances are also exploring hydrogels as bioink for bioprinting, a 3D printing technique that builds complex biological structures using living cells (Pu et al. 2024). While primarily used in medicine, bioprinting is emerging as a tool for soil research, enabling the creation of synthetic soil aggregates (SSAs) that mimic the porosity, permeability, and heterogeneity of natural aggregates (Smercina et al. 2022). SSAs are compatible with multi-omics and high-resolution imaging workflows and have been used to study microbial community distribution in structured habitats. This approach holds promise for exploring microbial spatial organization, soil carbon cycling, biogeochemical processes, and testing ecological theories such as niche theory, neutral theory, and modern coexistence theory (Baran et al. 2015, Laland et al. 2016, Smercina et al. 2022).

### Microfluidics

Microfluidic devices primarily come in two designs: channel-based and droplet-based, each with distinct applications. Droplet-based microfluidics are primarily used for fine-scale, reaction-based research, such as studying molecular interactions or single-cell responses to environmental stimuli (Ding et al. 2020). These droplets enable high-throughput experimentation, as robotics can generate droplets of varying sizes and treatments with high reproducibility (Ding et al. 2020). In contrast, channel-based microfluidics allow for larger organisms (i.e. plants) and their interactions with microbes or fungi to be studied within a single device (Noirot-Gros et al. 2020). These systems are 3D-printed chips that contain a network of micro to milli channels that allow for precise spatial and temporal control over various environmental conditions, such as pH, nutrient gradients, and moisture levels (Aleklett et al. 2018). They are equipped with sampling ports, making them suitable for -Omic approaches, and their transparent design is compatible with high-resolution microscopy techniques (Granzow et al. 2017, Aufrecht et al. 2018). These transparent 3D chips accommodate larger organisms (i.e seedlings) and can replicate specific soil physical properties by adjusting the material texture or channel design. This makes them ideal for visualizing and studying micro-scale interactions between plants and microorganisms (Zhu et al. 2022, p. 202).

Microfluidics devices that can recreate soil’s physical structure, including particle size and pore networks surrounding the roots are referred to as Rhizosphere-on-a-Chips (RoCs), and have enabled new ecology studies on how soil properties influence chemical heterogeneity and their subsequent impact on plant-microbe interactions. For example, RoCs have been used to study plant root exudation heterogeneity as the chip allows for the unique application of spatial metabolomics, the combination of metabolomics with spatial data to assign a location of the concentrations of a given metabolite (Aufrecht et al. 2022). RoCs have also been used in combination with other optical imaging techniques, with numerous applications ranging from root colonization dynamics to environmental stress response (Massalha et al. 2017, Stanley et al. 2018, Aufrecht et al. 2022). These applications could be extended to investigate rhizosphere biogeochemical cycling (Aufrecht et al. 2022, Agarwal et al. 2024), and ecological resilience, including how microbial communities and plants respond to environmental perturbations, recover from disturbances, and maintain functional stability under stress (Stanley et al. 2016, p. 201).

The extensive range of visual reporters that can be encoded in engineered microbes makes microfluidics compatible with a variety of synthetic biology applications. RoCs are a powerful platform for studying engineered plant-microbe interactions via metabolite diffusion, synthetic cell-cell surface interactions, chemotactic responses, and engineered HGT, in which cargo delivery efficiency is dependent on cell proximity (Fig. 3C) (Leal-Alves et al. 2024). They also can be useful to inform on gene circuit stochasticity and serve as a platform to troubleshoot biosystem design parameters, such as optimization of synthetic root exudate secretion (Fig. 3D).

While RoCs can be an excellent tool for investigating soil’s physical structure and their effect on various environmental phenomena, they have limitations. Microfluidic chips are often made of synthetic materials, such as polydimethylsiloxane (PDMS), which do not replicate the inorganic mineral content in natural soils (Aleklett et al. 2018). These materials, while convenient as they are transparent, moldable, and considered inert, can affect the results from RoC experiments as they do not replicate the chemistry of natural soil particles, including their capacity for nutrient retention and ionic exchange (Aleklett et al. 2018). The absence of these intrinsic properties, such as cation exchange capacity (CEC), can restrict studies on how nutrient exchange and organo-mineral interactions influence the behavior of plants and microbes. Moreover, the fabrication of RoCs​​ is complex and can be costly depending on different flow designs and fabrication materials, which limits throughput of combinatorial experiments that test different microbial communities, plant genotypes, and soil types (Zhu et al. 2022, Allan et al. 2024). Finally, RoCs size limits the scope of experiments, as larger plants can only be studied during early growth stages before outgrowing the device’s physical dimensions (Noirot-Gros et al. 2020). Although larger standardized devices for plant growth are being developed, their designs have not incorporated soil physical structure at larger scales (e.g. soil horizons) (Novak et al. 2024a). Therefore, future advancements in RoCs and other microfluidic systems should focus on expanding their designs to mimic regions ranging from micrometers to centimeters, incorporate scale-up designs that replicate natural biogeochemical gradients that develop with soil depth, and leverage emerging 3D-printing materials, surface-modification techniques (e.g. altering charge and hydrophobicity), or mineral coatings to better replicate natural soil chemistry (Alzahid et al. 2018, Wolf et al. 2018, Li et al. 2020, Sivaprakasam Padmanaban et al. 2022).

### Greenhouse-scalable soil models

Throughout this review, we have examined soil systems that replicate either the chemical or physical characteristics of natural soils. However, these systems are primarily designed for laboratory settings and for studying processes at small scales. A major challenge in ecology and synthetic biology is upscaling, translating insights from lab studies to greenhouse and field environments to accommodate larger plants, more complex environmental fluctuations, and longer time scales. In the next section, we review synthetic soils that have been used in larger growth chambers or greenhouses or that have the potential to be scaled up for these applications. Our focus will be on silica-based synthetic soils and pure soil amendments.

#### Silica-based artificial soils

Another approach to creating synthetic soils involves developing an inorganic base composed of key soil components, such as monodisperse quartz (SiO₂) particles of different sizes, clay minerals, calcium carbonates, and charcoal. The mix of inorganic materials can be tailored to achieve specific soil characteristics, including particle size distribution, mineral composition, and pH, which are essential for mimicking a natural soil environment. OM can be introduced in several forms: a liquid nutrient medium, a complex mixture of dissolved organic carbon (DOC), such as humic acids and malt extract, or sterile manure and peat, depending on the desired balance between nutrient control and realism. To replicate the three-dimensional structure characteristic of natural soils, the synthetic soils can be aggregated through both physical and chemical methods. Physical aggregation methods include wet-dry or freeze-thaw cycles, which promote natural compaction. Alternatively, chemical aggregation can be achieved through the incorporation of biopolymers (e.g. exopolysaccharides like xanthan gum), which enhance structural stability and soil cohesion (Guenet et al. 2011, Del Valle et al. 2022). These strategies create a functional soil system that closely resembles natural soils' structure and chemical properties.

Silica-based artificial soils have been primarily used in microbial studies to examine various factors influencing microbial activity and metabolism. These studies include investigating the effect of mineral composition on microbial activity and OM turnover, as well as the influence of particle size, mineral composition, and charcoal on microbial community structure (Ding et al. 2013, Pronk et al. 2013, Hemkemeyer et al. 2014, Vogel et al. 2014). Researchers have also explored microbial responses to environmental stressors, including the impact of contaminant addition on microbial communities and competitive interactions among microbial populations (Ellis 2004, Babin et al. 2014). Moreover, recent studies highlight the role of soil properties in facilitating microbe-microbe signaling, demonstrating how soil’s physical and chemical characteristics influence microbial communication mediated by diffusible chemicals (Del Valle et al. 2022). The rhizosphere has also been a focus of interest, with research showing that mineral composition plays a crucial role in determining microbial colonization patterns around plant roots (Whitman et al. 2018). These studies have addressed both transient microbial dynamics and long-term processes, such as aggregate formation, which have been observed over periods of up to two years following microbial inoculation (Pronk et al. 2017).

**Figure 4:**
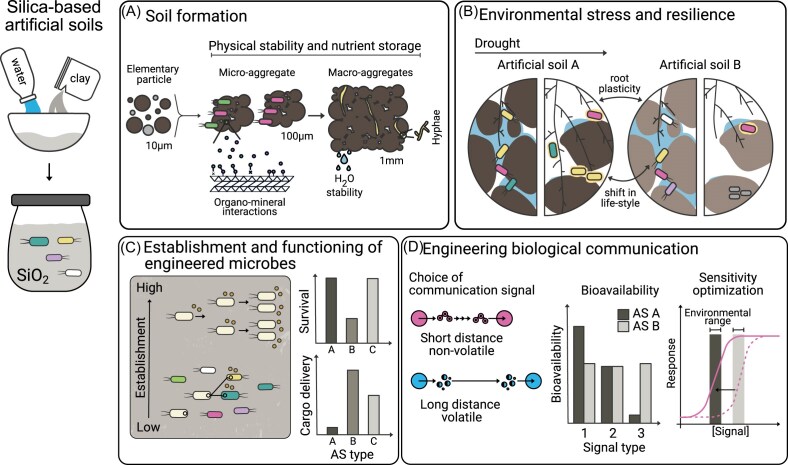
Silica-based artificial soils for probing microbially mediated soil processes. A) Silica-based artificial soils (AS) enable long-term incubations to assess microbial contributions to soil aggregation and structural development. B) Hydration levels can be precisely controlled to examine plant and microbial responses to environmental stress, including shifts in microbial life strategies and root plasticity. C) AS can be used to understand how well an engineered microbial ‘chassis’ establishes under different artificial soil types and how well it performs its engineered functions, for example, transfer of genetic cargo through HGT. D) AS can be used to determine what type of signaling performs best under different soil properties, for example, the type of communication signal (volatile vs. non-volatile), the bioavailability of the signal, and to tune the sensitivity of receiver cells to sense the signal under environmentally relevant conditions.

Silica-based artificial soils can be useful for answering similar questions as other synthetic soil systems, particularly when coupled with omics approaches, but are especially valuable for studying how plants and microbes modify soil properties, including changes in OM composition, soil aggregation, and mineral weathering (Fig. 4A) (Rabbi et al. 2024). These artificial soils can be also used to investigate community assembly and succession patterns following disturbance events, showing how pioneer species facilitate the establishment of later colonizers through environmental modifications. Additionally, they can provide insights into carbon allocation from plants to mycorrhizal fungi and the subsequent effects on soil aggregate stability, carbon sequestration across different soil fractions, and dynamics of OM stabilization. Similarly, any engineered organism designed to influence soil properties can be studied using this system. For example, engineered root-driven modification of soil environments and engineered microbes capable of producing different types of exopolysaccharides that can alter soil water retention and aggregation in response to environmental stress (Fig. 4B) (Rütering et al. 2017). Silica-based artificial soils can also be used to gain mechanistic insight into which soil factors influence the establishment of engineered microbes, thereby informing the selection of appropriate microbial ‘chassis’ for specific applications. For example, if the goal is for a plant growth-promoting bacterium to persist in the rhizosphere, high establishment efficiency may be desirable. In contrast, if the objective is to transfer a beneficial function to members of the native microbiome, the donor strain may require only transient establishment but efficient cargo delivery (Fig. 4C) (Albright et al. 2022). For applications focused on functional transfer to native communities, the compatibility of artificial soils with omics approaches also makes it well-suited for studying strains engineered with barcoding systems, such as those designed to track HGT using synthetic catalytic RNAs (Kalvapalle et al. 2026;). Furthermore, as mentioned above, these systems are well suited for characterizing biological communication, including plant-microbe and microbe-microbe interactions mediated by diffusible or gaseous signals. Biosensors designed to detect such communication can be evaluated to determine how different soil properties affect signal bioavailability and how biosensor sensitivity must be tuned accordingly (Fig. 4D).

Soil formation is driven by five key factors, including parent material, climate, biota, topography, and time, known as the CLORPT theory. While silica-based artificial soils provide valuable research opportunities, they cannot fully replicate the long-term development of natural soils influenced by the CLORPT factors (Eger et al. 2021). For example, organo-mineral interactions, which play a crucial role in soil structure and function, can take decades to form. Additionally, the complex interactions between vegetation, climate, parent material, and topography in natural environments are difficult to recreate in synthetic soils. To overcome some of these challenges, researchers have attempted to obtain the mineral fraction by directly deconstructing field soils instead of purchasing pure silica and minerals. For instance, an artificial soil designed to mimic rice field soil was created by extracting the mineral fraction, humic substances, and plant residues from natural soil, which were then remixed with an isolated microbial inoculum to study methanogenic OM decomposition (Maeda et al. 2020).

Another limitation of silica-based artificial soils is their opacity and autofluorescence, which makes them incompatible with optical techniques. This lack of transparency restricts high-resolution spatial studies and real-time visualization of microbial processes. Consequently, most techniques used with these soils require disruptive sampling methods, such as omics analyses. However, they remain compatible with certain non-destructive approaches, including the analysis of microbial respiration, biogeochemical gas fluxes, and the use of biosensors that rely on indicator gases (Guenet et al. 2011, Cheng et al. 2018).

Future improvements for silica-based artificial soils include expanding the recipe to include other types of minerals as base. Additionally, there is a need for a broader range of standardized OM formulations that can be added to the inorganic base. These formulations should encompass major classes of biochemically relevant compounds, including lipids, polysaccharides, lignin derivatives, and other recalcitrant organic fractions, beyond the commonly utilized water-soluble constituents (Argiroff et al. 2019).

Production of these artificial soils should also be scaled up for use in growth chambers and greenhouse studies. However, further testing is needed to assess their tensile strength and aggregate stability to ensure they provide long-term support for plant growth. Additionally, when scaling up production, it is important to implement safety measures to minimize exposure to silica nanoparticles, which can pose health risks if not properly managed (Liu and Sayes 2022).

### Amendments

Because greenhouse studies require substantial amounts of soil substrate, scientists have primarily focused on using commercially available soil amendments that are known to sustain plant growth under controlled conditions. While such materials are typically mixed with field or potting soil to improve specific properties (e.g. aeration, drainage, nutrient content), in this context we refer to their use as stand-alone substrates. That is, amendment-derived materials are used as the entire growth matrix to create a homogeneous synthetic soil, for example, pots filled with pure perlite, sand, or calcined clay rather than soil amended with these components. These amendments can be classified into two main categories: inorganic and organic substrates. Inorganic substrates include materials such as sand, vermiculite, calcined clay, perlite, and biochar. Organic substrates, on the other hand, consist of compost, peat, and woody residues. All these materials originate from natural sources and may undergo industrial processing before use to improve specific properties (Savvas and Gruda 2018).

Each substrate type offers distinct advantages and disadvantages. Sand and perlite have low nutrient-holding capacities but are highly effective in improving drainage and aeration (Wesström and Messing 2007). In contrast, organic materials generally offer better nutrient and water retention capacities; however, they display greater chemical variability, which restricts precise control of their individual physicochemical properties. For example, bark may contain fluctuating levels of terpenes and polyphenols, affecting plant growth and microbial activity (Del Valle et al. 2022). Efforts have been made to modify amendment properties to examine their effects on plant-microbe interactions. For example, sphagnum peat moss compost has been washed to reduce nutrients and then replenished with controlled additions, or milled to decrease pore size and examine its impact on plant growth (Masters-Clark et al. 2020).

**Figure 5:**
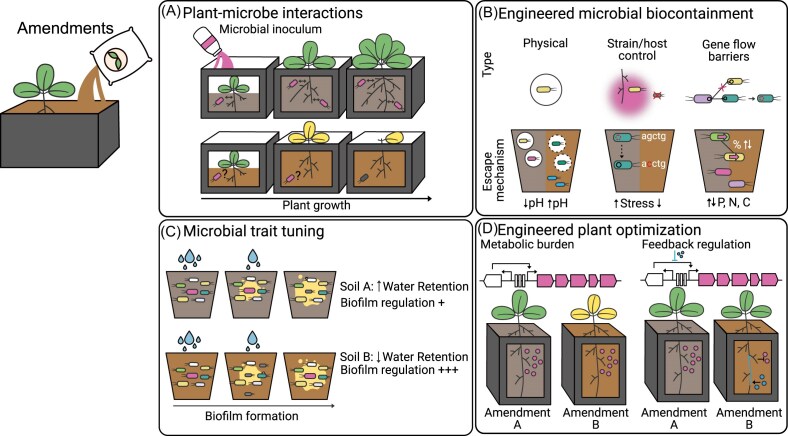
Soil amendments as bulk materials to investigate plant–microbe interactions. A) Soil amendments can support larger plant growth, facilitating longer-term experiments to evaluate the effects of adding a ‘soil probiotic’ containing a microbial inoculum of plant growth–promoting bacteria. B) Different microbial biocontainment strategies can be evaluated in amendments, including physical containment (e.g. encapsulated cells), host-controlled containment (e.g. dependence on root exudates), and gene flow barriers, as well as the rates and escape mechanisms that bypass engineered biocontainment strategies. C) Microbial traits can be tuned across different soil amendments. For example, biofilm production that enhances drought protection can be modulated depending on soil water retention properties. D) Engineered plants can be prototyped to determine how different amendments affect metabolic burden and whether they trigger feedback regulation.

Small-scale experiments using soil amendments are commonly employed to evaluate microbial metabolic capacity and shifts in microbial diversity in response to amendment addition. However, plant scientists and ecologists often use amendments without mixing them with field soil or commercial potting media in greenhouse-scale experiments due to their commercial availability and scalability. Because these materials can be purchased in bulk, they are well suited for high-throughput experiments requiring many treatments or replicates, as well as for studies involving larger or more mature plants that cannot be accommodated in smaller-scale systems such as microfluidic devices or TS. These amendments have been widely used to study the effects of environmental perturbations (e.g. elevated CO₂, temperature, precipitation) and assess ecological resilience (Bible et al. 2016, Appidi et al. 2024). To continue addressing ecological questions at a greenhouse scale, experiments should test how soil amendments affect plant and microbial responses to environmental feedback, and include multi-trophic interactions (e.g. nematodes, protists, microarthropods) to reveal controls on soil food webs and energy flow. Plants grown in different amendments can also be used to test plant growth promotion following the introduction of inoculants or ‘soil probiotics’ containing plant growth promoting bacteria across different plant developmental stages (Fig. 5A) (Moore et al. 2022).

Engineered microbes can be used to understand the effects of introducing different amendments to an ecosystem. For example, biosensors have been used to investigate how biochars produced from various feedstocks and pyrolysis temperatures can influence microbe-microbe communication mediated by small molecules (Masiello et al. 2013, Gao et al. 2016). Alternatively, microbial engineering can be intentionally designed so that the soil amendment functions as a prebiotic, creating a selective advantage for the engineered microbe to establish and persist in the soil or colonize plant roots. Amendments can also be used to shift bacterial community composition and abundance by incorporating a nutrient source that engineered specific members of the community can preferentially catabolize. Conversely, engineering bacteria to depend on a specific nutrient source, along with other approaches such as encapsulation or gene flow barriers, can serve as biocontainment strategies. Synthetic soils are a platform to study the mechanisms by which engineered strains persist, are constrained, or potentially escape in environmental contexts (Fig. 5B) (George et al. 2024).

Amendments can serve as inducers to trigger gene expression in engineered microbes, driving processes such as contaminant degradation, biocontrol against plant pathogens, or plant growth promotion through nutrient cycling (Fig. 5C). Similarly, plant engineering can be intentionally designed so that the soil amendment triggers the production of a metabolite of interest. Amendments as synthetic soils can be used to prototype metabolic burden under different amendment concentrations and to evaluate feedback regulation of the engineered metabolic pathways (Fig. 5D).

In addition to the variability in the composition of certain materials, some amendments are incompatible with microscopy and can be challenging to use for omics analyses. Their high sorptive nature makes extracting biological materials, such as DNA, proteins, and other biomolecules, difficult, which can introduce biases in the analysis. This complicates the use of omics techniques and may result in the loss of microbial diversity by negatively affecting the isolation of microorganisms.

These naturally heterogeneous materials can become even more variable, even when sourced from the same location, due to seasonal changes and slight differences in secondary processing methods. To improve reproducibility across research groups, it is essential for researchers to clearly describe not only the origin of the material but also its physicochemical properties. Utilizing standardized methods for characterizing these materials, such as measuring water-holding capacity, will further enhance consistency across studies (Barrett et al. 2016).

## Discussion

Synthetic soils are standardized systems that mimic an aspect of soils to isolate, control, and interrogate specific soil processes under reproducible conditions. They are designed to enable mechanistic understanding of the soil drivers that regulate biological and biogeochemical processes, and can help address ecological questions, such as linking microbiome structure to ecosystem function and test the performance of engineered plants and microbes under various environmental conditions. While each soil system described above has distinct strengths, limitations, and use cases, there is significant potential to integrate these systems to capture higher-complexity dynamics and tackle more challenging research questions (Table 1). For example, combining standardized chemical environments such as SESOM or MIMs with synthetic physical systems like transparent or silica-based soils could enable researchers to simultaneously control chemical composition and physical structure, offering new insights into soil-plant-microbe interactions under realistic yet reproducible conditions. Moreover, future synthetic soil research will benefit from studies that directly compare the performance of different synthetic soils systems when testing a single ecological hypothesis or engineered system (Novak et al. 2024b). Direct benchmarking experiments across soil systems will be useful to determine when simplified platforms faithfully reproduce biological phenomena observed in field soils.

**Table 1:** Growth system overview and use cases. Overview of the chemical, physical, and greenhouse scalable synthetic soils, highlighting their advantages, and limitations in the context of ecological experiments and synthetic biology applications.

Growth system	Advantages	Disadvantages	Technique compatibility	Science areas
Agar^†^	-Simple to sterilize -Reproducible -Easy to control variables -Interaction zones can be monitored and targeted (Basili et al. 2023) -Easy to extract metabolites -Easy to image	-Lacks soil physical and chemical complexity -Alters plant and microbial growth	-Omics* -Microscopic image Analysis** -Proximal imagery	-Plant (early development)-microbe interactions -Microbe-microbe interactions -Plant (early development) engineering -Single microbe/microbiome engineering
Soil-extracted solubilized organic and inorganic matter (SESOMS)	-Provides environmentally relevant organic matter chemistry (Manktelow et al. 2021) -Can retain some natural soil properties and microbes	-Less reproducible -Difficult to fully characterize	-Omics -Analytical chemistry & spectroscopy*** -Isotopic labelling -Proximal image analysis	-Microbe-microbe interactions -Single microbe/microbiome engineering
Metabolomics-based medium (MIMs)	-Can study chemical interactions without physical interference (Mehmood et al. 2019) -Environmentally derived metabolites allow for greater “realism” in experiments (Swenson et al. 2018)	-Lacks physical soil properties -Lacks some of the complex chemistry found in natural soils	-Omics -Analytical chemistry & spectroscopy -Isotopic labelling (Sharma et al. 2020) -Proximal image analysis	-Microbe-microbe interaction -Single microbe/microbiome engineering
Transparent soils (TS)	-Easy to image -Provides physical structure (Lozano-Andrade et al. 2023)	-Lacks relevant chemistry -Difficult to scale up -Can lack environmental relevance	-Proximal image analysis -Analytical chemistry & spectroscopy -Isotopic labelling -Omics	-Plant (early development)-microbe interactions -Microbe-microbe interaction -Plant (early development) engineering -Single microbe/microbiome engineering
Rhizosphere on a Chip (RoC) devices	-Easy to image -Provides insight into root based interactions (Mafla-Endara et al. 2021)	-Only µm-mm scale interactions -Lacks relevant chemistry	-Analytical chemistry & spectroscopy -Isotopic labelling -Microscopic image analysis -Proximal image analysis	-Plant (early development) -microbe interactions -Microbe-microbe interaction -Plant (early development) engineering -Single microbe/microbiome engineering
Silica-based Artificial Soils	-Reproducible -Can vary soil properties (Schabl et al. 2020 )	-Difficult to scale up -Can be difficult to compare to environmental conditions	-Omics -Analytical chemistry & spectroscopy -Proximal image analysis -Computer tomography	-Plant (early development)-microbe-soil interactions -Microbe-microbe interaction -Single microbe/microbiome engineering
Amendments (e.g. calcined clay/perlite)	-Simple to sterilize -Reproducible -Provides physical structure (Luecke et al. (2021) -Commercially available	-Difficult to extract metabolites -Homogeneous soil structure	-Proximal image analysis -Omics -Analytical chemistry & spectroscopy -Computer tomography	-Plant (mature) -microbe-soil interactions -Plant (mature) engineering -Single microbe/microbiome engineering
Field Collected Soils^†^	- Reflects natural soil properties (Xu et al. 2021)	-Difficult to extract metabolites -Cannot control nutrients -Cannot control soil structure -Difficult to sterilize -Not reproducible	-Omics -Genetic engineering -Analytical chemistry & Spectroscopy -Proximal image analysis -Computer tomography	-Plant (early development and mature)-microbe interactions -Microbe-microbe interactions -Plant (early development and mature) engineering -Single microbe/microbiome engineering

† Note that the agar and field-derived soils are not a part of the synthetic soils, rather, serve as the extremes two ends of the spectrum, one a fully synthetic growth media versus field soils.

* Omics: Includes the utilization of all forms of omic data (metabolomics, transcriptomics, metagenomics, etc.) as well sequencing of barcoded genetic mutants and other engineered strains.

** Microscopic Image analysis: Includes the use of Biorthogonal noncanonical amino acid tagging (BONCAT), fluorescent reporters, light and electron microscopy, whereas proximal image analysis includes hyperspectral imagery (HSI), dyes/staining, computer vision and machine learning for analyzing imagery taken from different synthetic soil systems.

*** Analytical Chemistry & Spectroscopy includes Gas Chromatography-Mass Spectrometry (GC-MS), Liquid Chromatography-Mass Spectrometry (LC-MS), Laser Induced Breakdown Spectroscopy (LIBS), Fourier Transform Infrared (FTIR) Spectroscopy, and other forms of chemical and spectroscopic analysis techniques.

Beyond integrating existing systems, there is also an opportunity to develop hybrid soil platforms that incorporate engineered smart materials capable of monitoring environmental conditions in real-time. The new materials could transform synthetic soils from static testbeds into dynamic platforms that can monitor the environmental changes. For example, pH-responsive materials embedded within synthetic soils have been used to resolve microscale pH gradients that emerge along growing roots, revealing how localized acidification alters nutrient availability and microbial colonization patterns (Patko et al. 2024). Similarly, developing moisture-sensitive materials to use in combination with synthetic soils could help track transient wetting-drying cycles and spatial moisture gradients, and provide mechanistic information on how hydration dynamics regulate plant and microbial metabolic activity, community turnover, and stress responses (Barnard et al. 2020). For example, incorporation of moisture sensitive materials could be used to study pulse-driven processes such as the Birch effect, linking rapid rewetting events to short-lived bursts of microbial activity and nutrient mineralization.

Advances in imaging and AI are also opening new possibilities for high-resolution, high-throughput analysis of soil-plant-microbe interactions in synthetic soils. Image-based chemical sensing and growth monitoring using RGB and hyperspectral cameras, combined with deep-learning algorithms like U-Net and YOLO, enable automated detection, classification, and segmentation of spatial, temporal, and chemical dynamics within complex systems (Massalha et al. 2017, Liu et al. 2021, Mafla-Endara et al. 2021, Bai et al. 2023, Diwan et al. 2023). When these AI advances are integrated with image-based phenotyping facilities [e.g. Advanced Plant Phenotyping Lab (APPL) (https://www.ornl.gov/appl), Ecotron Hasselt University (https://www.uhasselt.be/en/instituten-en/cmk-centre-for-environmental-sciences/infrastructure/ecotron)] equipped with electroptical techniques (multi and hyperspectral imagery, chlorophyll fluorescence cameras, LiDAR, etc.), and experiments are conducted in synthetic soils, they enable the integration of ground truth data with imagery for enhanced detection of plant disease, stressors, and plant-soil-microbe interactions, while providing mechanistic interpretation of how soil properties drive these responses (Pieruschka and Schurr 2019, Hall et al. 2022, Ma et al. 2022, Feyissa et al. 2025). For example, Feyissa et al. (2025) utilized the high throughput plant phenotyping facility APPL at Oak Ridge National Laboratory to look for genes of interest involved for enhancing plant photosynthetic efficiency and overall productivity through. Integrating such phenotyping facilities with synthetic soils could expand these types of studies allowing high throughput genetic screening of GWAS populations via plant growth metrics under controlled and systematically varied soil conditions. (Feyissa et al. 2025).

Recent advances in the standardization of collected field data can inform and constrain the design space of synthetic soils to make them more environmentally relevant. For example, the Molecular Observation Network (MONet) is a community-driven database for soil molecular and microstructure data developed by the Environmental Molecular Sciences Laboratory (EMSL) at the Pacific Northwest National Laboratory (PNNL). It provides open-source reference data for researchers of a variety of soil characteristics, including specialized data only accessible with advanced analytical techniques such as high-resolution molecular composition of SOM and three-dimensional soil microstructure [Molecular Observation Network (MONet) *|* Environmental Molecular Sciences Laboratory n.d.]. These datasets can be used to design synthetic soils that mimic the mineral composition, OM complexity, pore size distributions, and spatial heterogeneity observed in soil samples. In addition to MONet, similar databases maintained by government agencies and companies worldwide aim to provide standardized soil data, including texture, bulk density, soil pH, nutrient pools, root biomass distributions, and pedon descriptions with associated photos (Dangal and Sanderman 2020, Beem-Miller and Sierra 2023). These datasets can guide the selection of key soil characteristics to replicate in standardized soil platforms. For example, root biomass distributions can inform the design of RoCs, while reported nutrient pools can be used to constrain formulation of SESOMs and MIMs. Beyond informing initial design, well-characterized natural soils can also serve as benchmarks during development. In early stages of constructing a synthetic soil intended to reproduce functional or structural soil characteristics, side-by-side comparisons with a well-characterized reference soil can help evaluate whether the synthetic system captures relevant biological and physicochemical behaviors. Together, these resources enable the construction of soil-like systems that capture essential field-relevant features while maintaining experimental reproducibility and comparability across studies.

Another essential resource for guiding the development of synthetic soil systems is the availability of archived reference materials to use as standardized components in the development of synthetic soils. A good example is the repository of clays available for purchase at the Clay Mineral Society, which provides small quantities of reference clay minerals accompanied by detailed physical and chemical characterization, including mineralogical composition, CEC, and infrared spectroscopy (https://www.clays.org/). Similarly, the Edinburgh Standard Biochar set, which includes biochars produced from well-defined feedstocks under controlled and reproducible conditions, serve as reproducible reference materials to make synthetic soils for comparative studies (Mašek et al. 2018). For instance, standardized biochars from this set were used to investigate the mechanisms by which biochar affects microbial communication mediated by diffusible signaling molecules (Gao et al. 2016).

The complex biology underlying soil processes calls for greater interdisciplinary collaboration among ecologists, microbiologists, plant scientists, synthetic biologists, material scientists, and soil scientists, as each of these disciplines plays a key role in understanding the intricate dynamics of soils and the diverse organisms they support (Brevik et al. 2015). The development of more interdisciplinary training programs for students can also generate new approaches for the creation of these standardized soil systems (Lu et al. 2024). User facilities, such as EMSL and the Joint Genome Institute (JGI), can be another way to involve interdisciplinary teams. Facilities can provide funding opportunities for researchers and students alike to utilize expensive or large analytical equipment and expertise for projects that otherwise could be difficult to do at their home institutions.

In conclusion, standardized, manipulatable soil systems offer a versatile approach to advancing both fundamental and applied research in plant-microbe-soil interactions. These systems enable the disentanglement of highly variable edaphic factors from biotic interactions, allowing for the rigorous testing of ecological theories such as niche, neutral, and coexistence theory. They provide a powerful framework for investigating microbial community assembly within soils and plant roots, as well as for examining biodiversity-ecosystem function relationships. These insights can advance our understanding of microbial ecology, nutrient cycling, and plant health, ultimately informing sustainable ecosystem management practices and ecosystem restoration efforts (Le Roux et al. 2023, Wang et al. 2024). Furthermore, they are excellent platforms to fine-tune plant and microbial engineering using synthetic biology, facilitating design, test, build and learn (DBTL) cycles. They can function as testbeds during early R&D stages for developing engineered biofertilizers, biopesticides, and other agricultural and bioenergy innovations (Noirot-Gros et al. 2020). Additionally, these platforms can be used to evaluate genetically modified organisms for safety and performance before field deployment, ensuring that synthetic biology applications in agriculture and environmental management are safe and ready for regulatory approval (George et al. 2024, Chemla et al. 2025).
